# Concentration‐Based Analysis of Metal‐Induced Tau Fibrillar versus non‐fibrillar Aggregation: Implications for Neurotoxicity in Alzheimer's Disease

**DOI:** 10.1002/open.202400493

**Published:** 2025-03-24

**Authors:** Mahzad Irandoust, Afrooz Anbaraki, Zahra Dindar, Atiyeh Ghasemi, Ali Akbar Saboury, Saeed Rayati, Arefeh Seyedarabi

**Affiliations:** ^1^ Institute of Biochemistry and Biophysics University of Tehran Tehran Iran; ^2^ Department of Chemistry K.N. Toosi University of Technology, P.O. Box 16315-1618 Tehran 15418 Iran

**Keywords:** Metal ions, Tau protein, Fibrillation, Aggregation, Cytotoxicity

## Abstract

Tau protein aggregation is the most significant factor in Alzheimer's disease (AD) pathogenesis, and the accumulation of metal ions in the brain is considered a key factor in the development of this disease. Tau protein exhibits two distinct aggregate structures: fibrillar and non‐fibrillar aggregates. In this study, we conducted the first detailed study of the interactions of tau protein with three different concentrations of Zn^2+^, Cu^2+^, and Fe^3+^ions. Our findings demonstrate that low concentrations (0.01 mM) of these metal ions promote tau fibrillation, while higher concentrations (1 mM) induce non‐fibrillar aggregates. We have investigated the structural changes of tau by using advanced techniques such as SDS‐PAGE, DTNB, AFM, CD and fluorescence spectroscopy. At low concentrations, Zn^2+^ ions produced shorter fibrils, whereas Cu^2+^ and Fe^3+^ ions resulted in longer fibrils. CD showed increased β‐sheet structures with a decrease in random coil content. Interestingly, Cu^2+^ ions caused a significant decrease in neuronal viability. Our data highlights a new approach that illuminates the different ways in which the metal ions distinctively cause tau fibrillar versus non‐fibrillar aggregates, linked to neurotoxicity and neurodegeneration.

## Graphical abstract

Tau protein aggregation and fibrillation have been examined in relationship to three metal ions in this study. The research was conducted to see the effects of Zn^2+^, Cu^2+^ and Fe^3+^ ions at different concentrations on tau′s conformation, cytotoxicity in SH‐SY5Y cells, and free thiol levels. Results demonstrate that low concentrations of metals induce fibrillation, while higher concentrations lead to non‐fibrillar aggregates, with significant implications for neurodegenerative diseases.

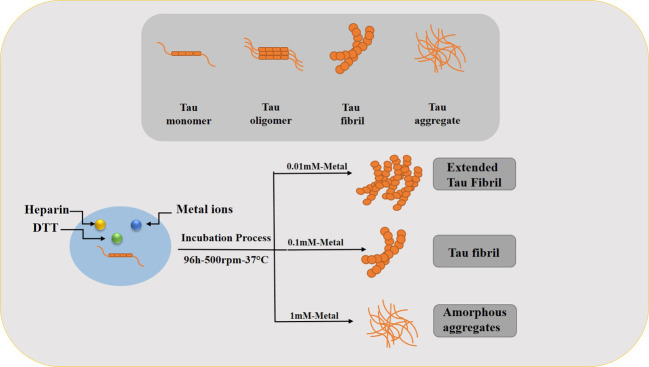

## Introduction

Alzheimer's disease (AD) is the most common neurodegenerative disorder associated with aging.^[1]^ The prominent clinical features of AD include significant loss of neurons as well as two distinct types of protein deposits in the brain: neurofibrillary tangles (NFTs) generated through hyperphosphorylation of tau protein and extracellular amyloid plaques caused by the fibrillation of amyloid beta (Aβ) peptide.^[2]^ Tau hyperphosphorylation is caused by an imbalance in the activity of a few protein kinases and phosphatases, which control the phosphorylation of tau proteins.^[3]^ When tau is phosphorylated abnormally, it separates from microtubules and gathers in the cytosol to form NFTs. These clumps of tau then cause neurodegenerative diseases like AD and other tauopathies.[^4^, ^5^]

One potent neurotoxic possibility that has been proposed to alter tau and Aβ aggregation is metal dyshomeostasis.^[6]^ Metal ions are vital to life and have significant functions in the human body. Metalloproteins, or proteins that bind metal, account for half of all proteins in nature.^[7]^ The metal cations, such as Zinc (Zn^2+^), magnesium (Mg^2+^), manganese (Mn^2+^), calcium (Ca^2+^), Iron (Fe^3+^, Fe^2+^) and Copper (Cu^2+^, Cu^+^), are essential for cell development, gene expression, signaling as a second messenger, and enzyme activity.^[8]^ Specifically, neuronal dysfunction leads to disruption of metal homeostasis and ultimately to the death of nerve cells.[^9^, ^10^] In typical circumstances, the levels of unbound metal ions within the brain remain minimal, exerting no influence on neurological operations. Conversely, the cerebral matter of individuals afflicted with AD harbors significant quantities of transition metal ions.^[11]^

Clinical research has shown that the concentrations of Zn^2+^, Cu^2+^ and Fe^3+^ ions are increased in the brain tissues of AD patients after death.[^12^, ^13^] On the other hand, it has been shown that Zn^2+^ deficiency can lead to cognitive problems, but taking Zn^2+^ supplements may improve brain function.^[14]^ The interaction of Zn^2+^ ion with tau protein has previously been attributed to residues Cys‐291 and Cys‐322, indicating that this site is the main site for high‐affinity binding of Zn^2+^ ion to tau and at low concentrations accelerates fibrillation of human tau protein by bridging between two cysteines under physiological reducing conditions.^[15]^ Zn^2+^ ion attach to tau and cause tau phosphorylation kinases and protein phosphatase 2 A to become inactive, hence promoting Tau′s hyperphosphorylation.^[16]^ However, recent preclinical studies indicate a possible phosphorylation‐independent mechanism involving the direct binding of Zn^2+^ ion to tau, which subsequently promotes its aggregation and modulates tau pathogenesis.[^17^, ^18^] Studies have shown that ROS forms in amyloid plaques and NFTs and that metals, such as Cu^2+^ ion, are able to accelerate the processes of oxidation and generation of ROS.^[19]^ Numerous comprehensive investigations have been conducted on the interactions between tau fragments and Cu^2+^ ion as documented in scholarly literature. These studies have shown that Cu^2+^ ion has a tendency to bind to the microtubule binding domains (R1, R2 and R3) of tau protein and thus induce structural changes in tau.[^20^, ^21^] The amino acid sequence of tau protein is relatively rich in histidine residues, and their imidazole side chain can be considered as a potential binding site for metal ions. Nearly all reported studies on the complex between tau proteins and metals have focused on the R1‐R4 domains in the microtubule binding region of tau protein.[^22^, ^23^] More than one Cu^2+^ ion binds to the R3 domain of the tau protein through two histidine residues, leading to a beta‐sheet enriched structural change in the protein. Additionally, the incubation of the R2 fragment of tau with Cu^2+^ ion results in the production of H₂O₂. In this process, Cu^2+^ ion is reduced to Cu^+^ion, and cross‐linking between peptide molecules occurs through the formation of disulfide bonds in oxidized cysteine.^[24]^ Cu^2+^ ion can interact with the N‐terminal region of tau protein, where histidine residues or amino groups can serve as specific binding sites.^[23]^ Interactions between Cu^2+^ ion and tau protein play a significant role in influencing the pathological changes observed in AD.^[25]^ Recent discoveries have shown that Cu^2+^ ion has a catalytic function in the process of tau protein aggregation, because it facilitates tau protein aggregation by inducing structural changes in tau located in the microtubule binding region.^[26]^ Additionally, it can induce tau protein hyperphosphorylation by activating the cyclin‐dependent kinase (CDK5/p25) complex and glycogen synthase kinase 3β (GSK3β).^[27]^ Adult neurodegenerative illnesses, like AD, are linked to an excessive buildup of Fe.^[28]^ Fe has a strong influence on the build‐up of tau protein brought on by oxidative stress.^[29]^ Investigations into tau‐laden filaments extracted from cerebral matter revealed that Fe nestled within ferritin might possess the ability to latch onto tau, igniting its clumping, and amplifying the attraction of Fe for phosphorylation.^[30]^ In a study examining the impact of Fe on kinase activity, it was suggested that Fe stimulates the activity of CDK5 but does not affect the activity of GSK3β.^[31]^ It has been shown that Fe has the ability to promote the aggregation of tau proteins by inducing a reversible structural alteration through its interaction with the threonine residue.^[32]^

Recent studies have shown that changes in the levels of various metals can affect the fibrillation process of tau protein; however, the exact mechanisms of these effects are still not fully understood.^[33]^ In this study, we investigated the effect of different concentrations of Zn^2+^, Cu^2+^ and Fe^3+^ ions on the fibrillation process of tau protein. Deeper knowledge about these effects can help to discover novel molecular pathways and devise treatments for neurodegenerative disorders. The present research is designed with the aim of testing a hypothesis about the pathological effects of essential metals on tau protein accumulation under in vitro conditions. For this purpose, methods such as ThT and ANS fluorescence tests, AFM, CD, MTT, DTNB and SDS‐PAGE were used. We aimed to answer the question of how different concentrations of metals can affect the fibrillation process of tau protein.

## Results and Discussion

### SDS‐PAGE Analysis of Tau Protein in the Presence of Different Metal Ions at Various concentrations

**Figure 1:**
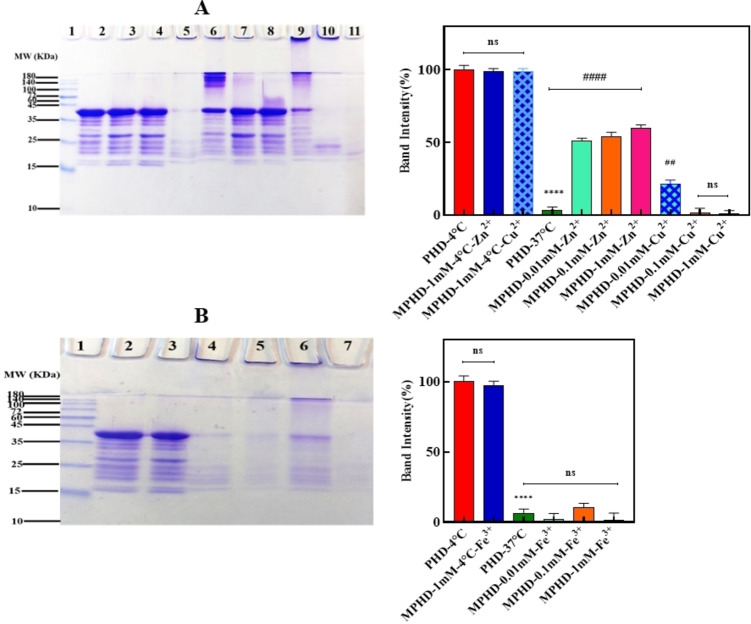
**SDS‐PAGE analysis of tau protein incubated with heparin, reducing agent, and different metal ions at various concentrations**. (A) Effect of Zn^2+^ and Cu^2+^ ions on tau fibrillation; 1: Protein marker, 2: not‐heated (protein in the absence of metal (negative control)), 3: not‐heated (protein in the presence of 1 mM Zn^2+^), 4: not‐heated (in the presence of 1 mM Cu^2+^), 5: Positive control treatment (protein in the absence of metal ions at 37 °C), 6: Protein treated with 0.01 mM Zn^2+^, 7: Protein treated with 0.1 mM Zn^2+^, 8: Protein treated with 1 mM Zn^2+^, 9: Protein treated with 0.01 mM Cu^2+^, 10: Protein treated with 0.1 mM Cu^2+^, 11: Protein treated with 1 mM Cu^2+^. (B) Effect of Fe^3+^ ions on tau fibrillation: Gel electrophoresis of tau protein treatments incubated with heparin and different Fe^3+^ ion concentrations; 1: Protein marker, 2: not‐heated (protein in the absence of metal (negative control)), 3: not‐heated (protein in the presence of 1 mM Fe^3+^), 4: Positive control treatment (protein in the absence of metal ions), 5: Protein treated with 0.01 mM Fe^3+^, 6: Protein treated with 0.1 mM Fe^3+^, 7: Protein treated with 1 mM Fe^3+^. Samples in lanes 5–11 in Figure 1A and samples 4–7 in Figure 1B were incubated. Statistical analyses was performed by t‐test and one‐way ANOVA followed by Dunnett's multiple comparisons test. ***P <0.001 and **P <0.01, significantly different from the negative control sample (PHD‐4 °C). #P <0.05, ##P <0.01, ###P <0.001, significantly different compared to the positive control sample. PHD: Protein + Heparin + DTT. MPHD: Metal + Protein + Heparin + DTT.

In AD, tau protein abnormally becomes phosphorylated and transforms into paired helical filaments.^[34]^ The recombinant tau protein, being intrinsically disordered, displays relative inertness with regards to spontaneous self‐organization, primarily attributed to the absence of necessary post‐translational modifications conducive to aggregation. Nonetheless, the fibrillation and aggregation of tau protein may be triggered by diverse factors, such as exposure to poly anions like heparin, mutations predisposing to aggregation, or protein truncations.^[35]^ Pretangle neurons collect sulfated glycosaminoglycans such as heparin and heparan sulfate, which themselves induce tau phosphorylation under experimental conditions and lead to tau aggregation.^[36]^ Tau monomers do not aggregate spontaneously within experimentally feasible time frames.^[37]^ Tau accumulation proceeds pathologically via a nucleation‐elongation process involving β structure formation.[^38^, ^39^] In the current study, DTT was used as a strong reducing agent to mimic the reducing environment found in natural neuronal cells. The results clearly demonstrated that under physiological pH, heparin and DTT, tau formed fibrils.^[17]^ Heparin and DTT are the main agents that induce tau protein into fibrillar structures.^[40]^ The tau protein (4R isoform) has two cysteine residues that engage in intricate inter/intramolecular disulfide interactions. The increase in intramolecular disulfide bonds leads to the formation of amorphous aggregates, preventing the development of beta structures and thereby hindering the assembly of tau protein fibrils. Additionally, the cysteine mutant of full‐length tau or of its truncated variant (spanning residues 297–391 of full‐length tau) has the capability to assemble into tau fibrils. It is suggested that intermolecular disulfide bridges contribute only to the early stages of tau fibril formation, accelerating the process. Therefore, in this study, to prevent intramolecular disulfide bonding and accelerate the in vitro formation of tau fibrils, heparin‐induced fibrillation of the 1 N4R tau isoform was conducted under reducing conditions using DTT, consistent with previous research.^[41]^ Tau aggregation can be influenced by metals such as Zn^2+^, Cu^2+^ and Fe^3+^, which have been shown to modulate kinases that phosphorylate tau, exacerbating tau pathology.^[24]^ Modifications of the tau protein in the presence of heparin, the reducing agent DTT, and metal compounds, demonstrated that the highest fibrillation was observed at the lowest concentration of metals (0.01 mM), while at the highest concentration of metals (1 mM), protein aggregation was noted. Low levels of Zn^2+^ ions have been shown to exacerbate fibril formation, while high levels promote aggregation.[^15^, ^42^] Low concentrations of Zn^2+^ ions are proved to be responsible for the aggravation of fibril formation, while high amounts are the causes of the aggregation. This can be due to a change in the Zn^2+^ ion binding sites with increasing metal concentrations.^[43]^ The SDS‐PAGE method allows one to study fibrillation, aggregation, and the formation of species with higher molecular weights in a protein samples.^[40]^ The tau protein has two kinds of aggregate structures, one being amorphous aggregates and the other being fibrillar species.^[17]^ The results of the electrophoretic evaluation of tau protein modifications demonstrated that metals led to the formation of cross‐links and the creation of high molecular weight species in tau protein. These high molecular weight species formed at the boundary between the separating and stacking gels with an approximate molecular weight of 100 to 180 kDa, and even higher molecular weight species were also formed in the well of the gel.^[44]^ In this study, tau protein was exposed to the metal compounds for 96 hours at 37 °C under shaking conditions. In the results of the tau protein evaluation (Figure 1A), we observed no changes since the monomeric tau band was maintained in the not‐heated sample (at 4 °C), both in the absence (lane 2) and in the presence of 1 mM Zn^2+^ and Cu^2+^ ions (lanes 3 and 4, respectively). Thus, a high concentration of the metals at 4° C did not affect the protein structure and was very similar to the negative control (the sample without metals in lane 2). As shown in lane 5, heparin and DTT in the absence of Zn^2+^ and Cu^2+^ ions led to the formation of cross‐linked species and higher molecular weight species in the treated tau protein. The intensity of the tau monomer protein band (positive control) has also decreased compared to samples without heat. The disappearance of the tau monomer has been mainly attributed to the formation of high molecular weight fibrils.^[45]^ Lanes 6 to 8 show the electrophoretic pattern of tau samples exposed to the Zn^2+^ metal compound (at three concentrations of 0.01, 0.1 and 1 mM, respectively). Lanes 9 to 11 depicted the electrophoretic pattern of tau samples treated with the Cu^2+^ metal compound at three different concentrations, namely 0.01, 0.1, and 1 mM, respectively. According to our results, incubation of tau with both Zn^2+^ and Cu^2+^ ions at a 0.01 mM concentration increased the formation of protein fibrillation and higher molecular weight species, as shown in lanes 6 and 9, respectively. Moreover, the tau protein monomeric band was less intense compared to the not‐heated control sample. Lanes 7 and 10, which correspond to samples of tau exposed to 0.1 mM Zn^2+^ and Cu^2+^ ions, respectively, show protein bands formed in the well region of the gel and at the border between the stacking and resolving regions of the gel. In the samples treated with Zn^2+^ ions, the intensity of the tau protein monomeric band was higher than the sample exposed to 0.01 mM Zn^2+^ ions and the positive control sample. On the other hand, in the sample treated with Cu^2+^ ions at a concentration of 0.1 mM, the intensity of the monomeric band decreased and became closer to the intensity of the positive control sample. In lane 8, which corresponds to tau sample treated with 1 mM Zn^2+^ ions, the intensity of the tau monomeric band was higher compared to the lower concentrations of this metal and was very similar to the non‐heated samples. This shows that this concentration can restore the altered form of tau protein to its normal state. But at a concentration of 1 mM Cu^2+^ (lane 11), a large decrease in the monomeric band intensity was observed, as well as high molecular weight proteins, which formed in the well region of the gel. According to Figure 1A, there was no significant difference between the negative control (not‐heated tau sample without metal ions) and the two not‐heated samples containing metal (P>0.05), but there was a significant difference between the negative control and the positive control (P<0.05). Comparing the samples treated with Zn^2+^ and Cu^2+^ ions with the positive control sample showed that there is a significant difference in all three concentrations of Zn^2+^ and in the low concentration of Cu^2+^ (P<0.05). But this difference was not seen in the two higher concentrations of Cu^2+^ (P>0.05). However, for the two higher concentrations of Cu^2+^, this difference was not observed.

The examination of Fe^3+^ ion's effect on tau aggregation showed that tau treated with heparin and the reducing agent DTT (lane 2), as well as 1 mM Fe^3+^ (lane 3), did not alter the intensity of the monomeric protein band. This indicates that a high concentration of Fe^3+^ ions at 4 °C did not affect the protein structure (Figure 1B). At lower Fe^3+^ concentrations (lane 4), however, protein aggregation occurred at the interfaces of the stacking and resolving region of the gel and within the wells of the gels. In conclusion, no significant difference was observed between the negative control and the Fe^3+^‐treated sample (at 4 °C), neither between the positive control, nor the different Fe^3+^ ion concentrations in lanes 5–7 (P >0.05).

#### ThT Analysis of Tau Protein in the Presence of Different Metal Ions at Various Concentrations

**Figure 2:**
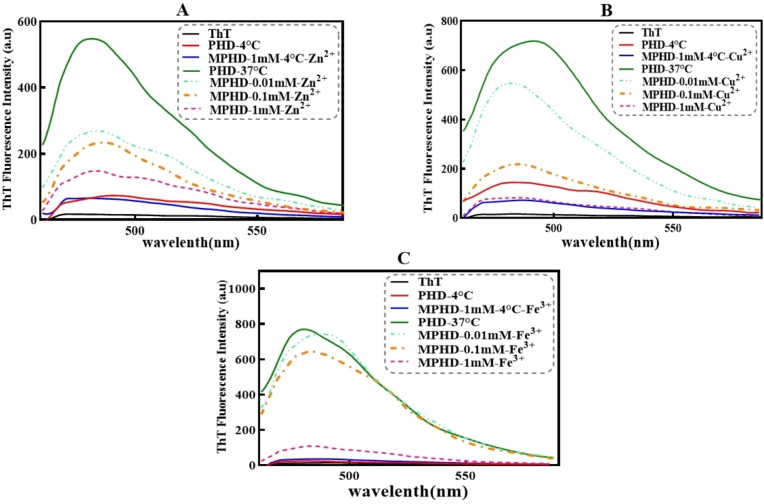
**ThT fluorescence data of tau incubated with different metal ions at various concentrations**. (A−C) ThT fluorescence emission data of tau incubated with Zn^2+^, Cu^2+^ and Fe^3+^ ions, at 0.01, 0.1 and 1 mM concentrations, respectively. PHD: Protein + Heparin + DTT. MPHD: Metal + Protein + Heparin + DTT. The samples, abbreviated as MPHD, were incubated at 37 °C for 96 hours at three different concentrations of various metal ions, along with the positive control sample.

The increase in the fluorescence intensity of ThT and ANS has been used in previous studies as an indication of tau fibrillation.^[46]^ In the present study, tau protein solutions at a concentration of 20 μM were prepared in 50 mM Tris buffer at pH 7.4 combined with 100 mM NaCl. Using these experimental set‐up conditions, the effects of different concentrations of metals on fibrillation and tau protein accumulation were detected by an increase in the fluorescence intensity. The ThT fluorescence results showed that the positive control samples and those treated with 0.01 and 0.1 mM concentrations of Zn^2+^, Cu^2+^, and Fe^3+^ ions in the presence of the reducing agent and heparin, exhibited increased fluorescence emission and amyloid fibril formation. In contrast, 1 mM of metals showed similar results compared to the negative control (Figure 2). As shown in Figure S1, a significant difference was found in both negative and positive controls and between the positive control and samples treated with different concentrations of the ions, except the sample treated with 1 mM Fe^3+^ (P <0.05). The enhancement in tau protein fibrillation was more pronounced for lower concentrations of all these metals.

#### ANS Analysis Of Tau Protein in the Presence of Different Metal Ions at Various Concentrations

**Figure 3:**
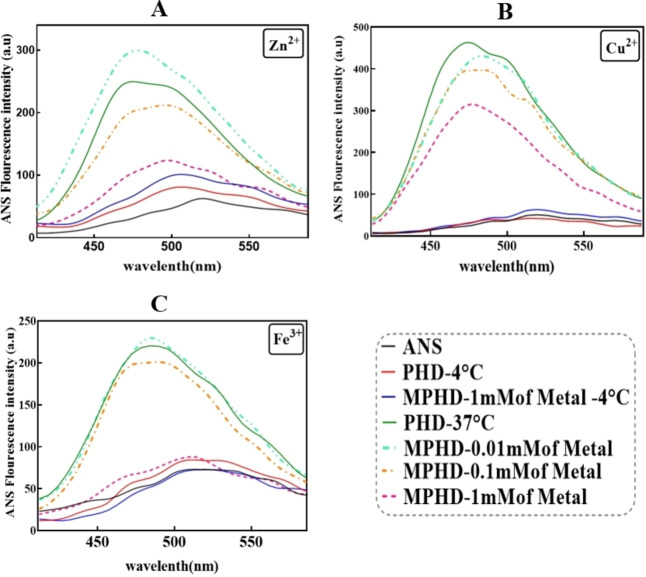
**ANS fluorescence data analysis of tau incubated with different metal ions at various concentrations**. (A−C) ANS fluorescence emission data of tau incubated with Zn^2+^, Cu^2+^ and Fe^3+^ ions, at 0.01, 0.1 and 1 mM concentrations, respectively. PHD: Protein + Heparin + DTT. MPHD: Metal + Protein + Heparin + DTT. The samples, abbreviated as MPHD, were incubated at 37 °C for 96 hours at three different concentrations of various metal ions, along with the positive control sample.

The structural changes in tau protein samples were measured by ANS spectroscopy.^[44]^ The ANS probe identifies hydrophobic regions on protein surfaces that get exposed due to misfolded conformations of the protein.^[47]^ ANS will fluoresce upon binding to hydrophobic regions of proteins, and thus measurement of its emission can give information on structural changes related to the protein hydrophobic patches and subsequent fibrillation.^[48]^ Tau protein solutions were prepared in 50 mM Tris buffer at pH 7.4 combined with 100 mM NaCl. The results from this study showed that the ANS fluorescence spectra related to the positive control sample was increased as well as those samples of tau incubated with low concentrations of Zn^2+^ ions, compared to the not‐heated sample, indicating amyloid fibril formation. As such, the highest emission was seen for tau treated with 0.01 mM Zn^2+^ ions, whereas tau treated with 1 mM Zn^2+^ ions, had an emission similar to the not‐heated sample. (Figure 3A) In Figure S2, it is described that there is a significant difference between the negative control and positive control and also between the positive control and Zn^2+^ incubated samples. For Cu^2+^ and Fe^3+^ ion treatments, higher fluorescence was also found for the lower concentrations at 0.01 and 0.1 mM compared to the 1 mM and not‐heated samples (Figs. B and C). The negative control was not different from the not‐heated samples containing Cu^2+^ or Fe^3+^ ions (P >0.05). However, significant differences were noted between the negative control and positive control (P <0.05), as well as between the positive control and samples treated with Cu^2+^ and Fe^3+^ ions, except for the 0.01 mM Cu^2+^ and Fe^3+^ concentrations and the 0.1 mM Fe^3+^ concentration (Figure S2). In this experimental study, the potential impact of metal ions on the fluorescence intensity of ThT and ANS was also examined. As a control, metal‐containing samples without the presence of the tau protein were tested under similar conditions, at three different concentrations. Following this, the measured fluorescence intensities of ThT and ANS for these samples were subtracted (Figure S3). It is noteworthy that there were no substantial differences between the fluorescence intensity of the blank samples and the ThT and ANS samples, used as the control.

#### Secondary Structure Analysis of Tau Protein in the Presence of Different Metal Ions at Various Concentrations

**Figure 4:**
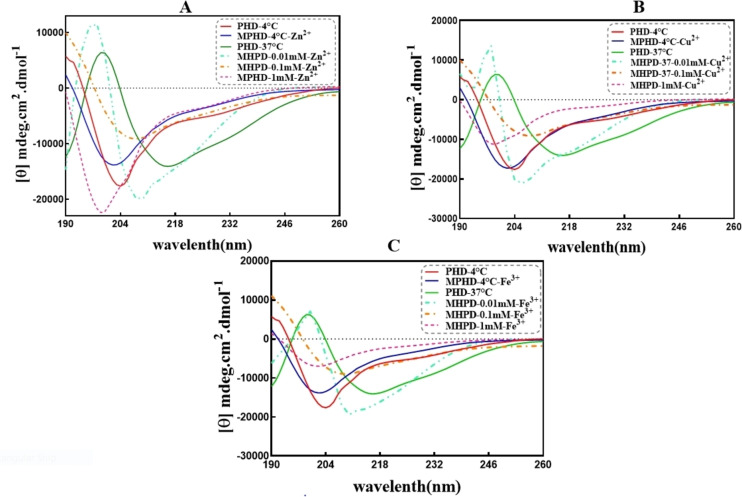
**Secondary structure analysis of tau incubated with different metal ions at various concentrations**. (A−C) CD spectra of tau incubated with Zn^2+^, Cu^2+^ and Fe^3+^ ions, at 0.01, 0.1 and 1 mM concentrations, respectively. PHD: Protein + Heparin + DTT. MPHD: Metal + Protein + Heparin + DTT. The samples, abbreviated as MPHD, were incubated at 37 °C for 96 hours at three different concentrations of various metal ions, along with the positive control sample.

Circular dichroism (CD) is a sensitive spectroscopic technique used to study the secondary structure and folding properties of proteins. One of the structural changes in tau protein during fibrillation is the transition of the native random coil structures to parallel β‐sheet content.[^40^, ^49^] The results of the CD spectra of tau treatments in the far‐UV region, showing the secondary structure of tau in the wavelength range of 190 to 260 nm, indicated that the not‐heated sample had a negative peak at around 204 nm (Figure 4).[^44^, ^50^] Since tau protein inherently lacks a well‐defined structure, its CD spectrum showed only a negative peak at 204 nm, indicating the presence of random coil structures.^[51]^ In the CD spectra of tau incubated with Zn^2+^, Cu^2+^, and Fe^3+^ ions under fibrillation conditions, a negative peak at 217 nm was observed, which is characteristic of β‐sheet structures.[^52^, ^53^] Therefore, the presence of heparin, DTT as reducing agent, and metals with tau at 37 °C led to the formation of β‐sheet‐rich structures and amyloid fibril formation.

The results of the studies indicated that pre‐fibrillar amyloid species typically adopt antiparallel β‐sheet structures, which transform into parallel β‐sheet structures in fibrillar species.[^54^, ^55^] A comparison of the secondary structure content of tau protein incubated with metal ions, using the BeStSel software to determine the percentage of each secondary structure in the spectra, showed that the percentage of β‐sheet structures in the positive control treatment increased compared to the not‐heated sample (negative control‐ protein in the absence of metal ions) and the sample in the presence of the 1 mM metal ions. The amount of random coil structures also decreased. For the metal‐containing samples, the percentage of β‐sheet structures increased, especially parallel β‐sheets, with the decrease of the metal ion concentrations compared to the not‐heated sample and even the positive control sample. In contrast, the percentage of random coil structure remained almost the same as that of the positive control sample. This result showed that the amount of β‐sheet structures increases with the decreasing concentration of metal ions (Tables S1, S2, and S3).

#### Studying the Morphological Alterations of Tau Protein in the Presence of Different Metal Ions At Various Concentrations

**Figure 5:**
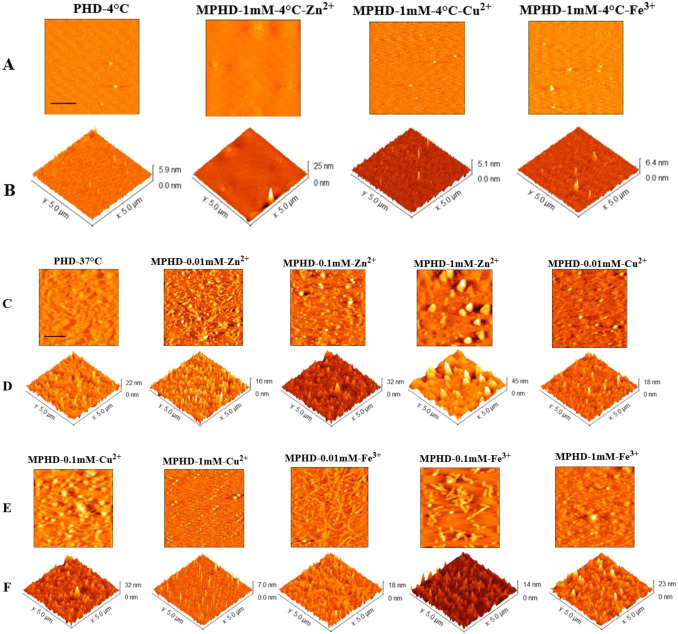
**AFM analysis of tau fibrillar samples and native tau aggregates**. (A, C and E) 2D‐AFM images of different tau samples. (B, D and F) 3D‐ AFM images of different tau samples. Images from left include: not‐heated control (without metal ions), not‐heated treated with 1 mM concentrations of Fe^3+^, Cu^2+^, and Zn^2+^, positive control (protein in the absence of metal ions), treatment with 0.1 mM concentration of Zn^2+^, treatment with 1.0 mM concentration of Zn^2+^, treatment with 1.0 mM concentration of Cu^2+^, treatment with 0.1 mM concentration of Cu^2+^, treatment with 1.0 mM concentration of Cu^2+^, treatment with 0.1 mM concentration of Fe^3+^ and treatment with 1.0 mM concentration of Fe^3+^. AFM images were collected after 96 hours of incubation. Scale bar in all images: 1 micron. PHD: Protein + Heparin + DTT. MPHD: Metal + Protein + Heparin + DTT.

Among important aberrant protein assemblages in tau protein in AD, there are two types: aggregates and fibrils. Aggregates are structures that are of a lower order, chaotic, and involve various units of tau proteins. By contrast, fibrils are long, orderly, fibrous structures often found inside cells in the filamentous shape; these consist of many tau proteins in a continuous, stable structure.^[56]^ Zn^2+^, Cu^2+^, and Fe^3+^ are examples of metals that can affect the stability and structure of fibrils as well as aggregates. To examine the structure and morphology of tau treated with the metal ions, the samples were incubated with 50 mM Tris buffer at pH 7.4, 100 mM NaCl and metal ions, and then examined using AFM. Figure 5 shows that the positive control samples and those treated with lower concentrations of metals exhibit mature fibril structures compared to the not‐heated samples, where no fibrillation was observed. The two‐dimensional images indicated that lower metal concentrations led to the formation of tau protein fibrils, while higher concentrations prevented the formation of fibrillar structures and instead directed the tau protein towards aggregate formation. The images revealed different polymorphisms induced by various metals. A study examining the effect of a 0.1 mM concentration of metals revealed that Zn^2+^ induces the formation of spherical oligomers, Cu^2+^ leads to the formation of large protofibrils, and Fe^3+^ results in the formation of small protofibrils.^[57]^ Additionally, a study on alpha‐synuclein protein showed that different metals are capable of creating polymorphic fibers.^[58]^ In another study on alpha‐synuclein, Fe^3+^ ions induced the formation of small protofibrils, while Zn^2+^ ions promoted the formation of spherical oligomers.^[59]^ Inquisitive studies have clearly illuminated that these three metal ions remarkably hasten the clumping of tau proteins, while concurrently amplifying the creation of tau oligomers.^[60]^ Although metals have different effects on the type and size of aggregates, the results of this study indicated that the concentration of metals also plays a crucial role in fibril formation. Analysis of the height distribution of the tau samples showed that the average height of monomers was about 1.8 nm, while in the fibrillar samples, it was approximately 14.51 nm. The increase in average height distribution was dose‐dependent, particularly notable at lower metal concentrations (Figure S4).

#### Evaluation of Neurotoxicity of Tau Protein in the Presence of different Metal Ions at Various Concentrations

**Figure 6:**
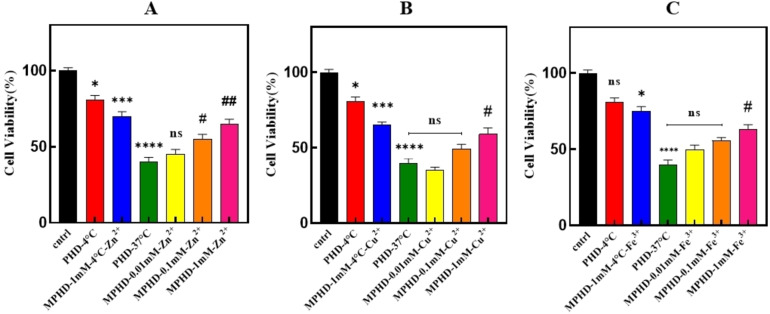
**Neurotoxicity assessment of tau protein incubated with various metal ions at different concentrations using the SH‐SY5Y cell line**. Statistical analysis was performed by t‐test and one‐way ANOVA followed by Dunnett's multiple comparisons test. ***P <0.001 and **P <0.01, significantly different from the negative control sample (PHD‐4 °C). #P <0.05, ##P <0.01, ###P <0.001, significantly different compared to the positive control sample. PHD: Protein + Heparin + DTT. MPHD: Metal + Protein + Heparin + DTT.

Accumulation of tau protein has been implicated in neurotoxicity, complicating cognitive impairments in AD.^[60]^ Based on the differences in fibril morphologies seen in this study, it was hypothesized that the differences could give rise to some variation in the levels of cellular toxicity. For example, spherical oligomers were seen to form in the presence of Zn^2+^ ions during the present study and although some of these oligomers can represent intermediates in the pathway to amyloid fibril formation, other oligomers may represent off‐pathway products, which could be toxic. Therefore, research was conducted into the toxic action of Zn^2+^, Cu^2+^, and Fe^3+^ ions by using the cell line SH‐SY5Y, which in the last decade has been very popular for a wide spectrum of neurotoxicity investigations due to its distinctive biological features, namely, that it can maintain either in a proliferative or differentiated cellular state upon the addition of specific media.[^61^, ^62^] The SH‐SY5Y cell line has been widely used by researchers to investigate the cytotoxic effects of metals on neuronal cells under neurodegenerative conditions like AD.[^63^, ^64^] However, the specific effects of variable Zn^2+^, Cu^2+^, and Fe^3+^ ions on neurotoxicity and morphological changes had not been sufficiently documented with appropriate models. In the present work, the determination of cell viability after 96 hours of incubation with cells treated with tau under fibrillation conditions in the presence of metal ions showed that the viability was higher in the case of not‐heated samples than the positive control and the metals‐treated samples (Figure 6). Moreover, the highest similarity in cell viability to the positive control was observed in treatments with 0.01 mM concentrations of metals. Conversely, treatments with 0.1 and 1 mM concentrations of metals showed more than 50 % cell viability, indicating their lower toxicity compared to the 0.01 mM concentration of the three metals. It is worth mentioning that the treatment performed with the 0.01 mM concentration of Cu^2+^ presented higher toxicity, presenting cell viability of less than 40 %. Amongst the papers relating to Aβ, one of them identified that the toxicity provided by Cu^2+^ ions is higher when compared to Zn^2+^ ions.^[65]^ Significant differences (P <0.05) were observed between the negative control (not‐heated sample without Zn^2+^, Cu^2+^ and Fe^3+^) and the not‐heated treatment containing 1 mM concentration of metals (at 4 °C). Comparisons between the positive control and tau treated with Zn^2+^, Cu^2+^, and Fe^3+^ metals showed significant differences (P <0.05). However, there was no significant difference, at P >0.05, between the positive control and the treatments with 0.01 mM of Zn^2+^. Conversely, the positive control maintained significant differences, at P <0.05, when compared to tau treated with 0.1 and 1 mM of Zn^2+^ ions. Furthermore, a significant relationship was observed between the positive control and treatments with 1 mM concentrations of Cu^2+^ and Fe^3+^ ions (P <0.05). However, no significant difference (P >0.05) was found between the positive control and tau treated with lower concentrations of Cu^2+^ and Fe^3+^ ions. The cytotoxic effects of metals in the absence of tau showed that the cell survival was more than 70 %. (Figure S5).

#### Assessment of Free Thiol Levels of Tau Protein in the presence of Different Metal Ions at Various Concentrations Using the Ellman's Reagent

**Figure 7:**
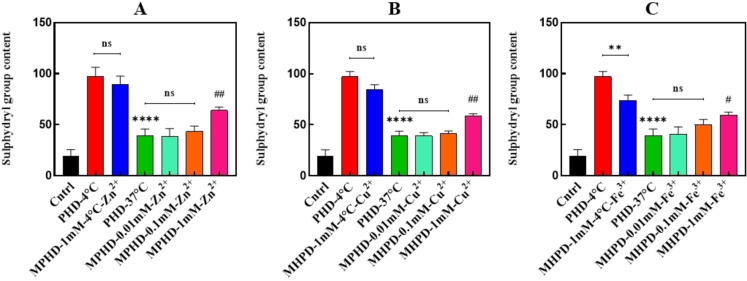
**Assessment of free thiol levels of tau incubated with different metal ions at various concentrations, using the Ellman's reagent (DTNB) colorimetric method**. Statistical analysis was performed by t‐test and one‐way ANOVA followed by Dunnett's multiple comparisons test. ***P <0.001 and **P <0.01, significantly different from the negative control sample (PHD‐4 °C). #P <0.05, ##P <0.01, ###P <0.001, significantly different compared to the positive control sample. PHD: Protein + Heparin + DTT. MPHD: Metal + Protein + Heparin + DTT.

The compound 5,5′‐dithiobis‐2‐nitrobenzoic acid (DTNB) is used to measure free thiol levels in proteins. Each molecule containing free thiols reacts with DTNB through a substitution mechanism. When DTNB binds to free thiols, it is converted into thionitrobenzoic protein (TNBP) and 5‐thio‐2‐nitrobenzoic acid (TNBA). Under neutral or alkaline conditions and in the presence of high levels of free thiols, this reaction produces an intense yellow chromophore. The absorbance of this chromophore can be measured at a wavelength of 412 nm, with a molar extinction coefficient of 14,150 M^−1^ cm^−1^, to quantify the levels of free thiols.^[66]^ In the context of tau protein interactions, disulfide cross‐linking plays a significant role in modulating structural assembly and stability. Free thiol groups in cysteine residues can participate in disulfide bond formation, driving intermolecular interactions and promoting the aggregation of tau molecules into oligomers and higher‐order structures.^[67]^ DTNB assays provide a sensitive method to monitor these processes by detecting changes in the availability of free thiols. The Tris‐NaCl buffer with a pH of 7.4 was prepared as the solvent for DTNB. After 96 hours of incubation of tau samples at a concentration of 20 μL, they were combined with DTNB at a molar ratio of 1 : 7. The samples were then kept in the dark for 90 minutes, and their absorbance was subsequently measured. The results indicated that in the not‐heated samples, the release of DTNB was high due to the presence of free thiol groups, whereas the positive control treatment showed significantly lower release of DTNB. As the metal concentrations decreased from 1 mM to 0.01 mM, the release of DTNB also decreased, with the 0.1 mM and 0.01 mM concentrations closely resembling the positive control treatment. Comparisons between the negative and positive controls revealed significant differences, as well as notable differences between the positive control and treatments containing metals at a concentration of 1 mM (P <0.05) (Figure 7).

## Conclusions

In the present study, the investigation into the effects of various metal compounds on tau protein fibrillation and aggregation was conducted in a comprehensive way. Given the two distinct aggregation structures in tau protein (fibrillar aggregates and non‐fibrillar aggregates), tau was exposed to increasing concentrations of metals in the presence of heparin and DTT for an incubation period of 96 h. Although previous studies had been conducted on the effects of metal ions on tau protein, however, non‐had investigated varying concentrations of Zn^2+^, Cu^2+^, and Fe^3+^ ions and their effects on tau protein, distinguishing between fibrillar and non‐fibrillar aggregate formation. Therefore, in this current study a novel approach was taken whereby varying concentrations of these metal ions had been studied, enabling the evaluation of their distinct effects on tau protein fibrillation versus aggregation. The results from this study confirmed that low concentrations of the three metal ions induced protein fibrillation, while higher concentrations resulted in the formation of non‐fibrillar aggregates. It was also noted that the presence of metal ions increased the number of morphological structures formed. This underlined the different effects of metal ions on tau protein conformation. It was also shown in cell experiments that such metal concentrations might reduce cell viability by showing the possible cytotoxicity of these metals under specific conditions. This study uniquely amalgamated a broad palette of analytical techniques in investigations into the interplay between tau protein and metal ions. The results of this present study clearly showed that metal ions have a great influence on both tau protein fibrillation and aggregation. The present data may pave a better path for effective therapies for neurodegenerative diseases. Future studies will focus on more detailed analyses of the long‐term effects of metal ions on tau protein and their interactions with cellular functions.

### Experimental Procedures

#### Materials

ZnCl_2_, CuCl_2_ and Fe_2_Cl_3_ were purchased from Merck. 5,5′‐Dithio bis(2‐nitrobenzoic) (DTNB) was obtained from Sigma‐Aldrich chemical company and dithiothreitol (DTT; CAS No:3483‐12‐3) was purchased from Sigma‐Aldrich. Luria Bertani (LB) broth was also obtained from the Merck Company. Heparin sodium (5000 IU, average molecular mass of 16 kDa) was purchased from the Iran hormone pharmaceutical Company. ArgPure nickel sepharose 6B fast flow and Isopropyl ß‐D‐1 thiogalactopyranoside (IPTG) were purchased from ARG biotechnology and Sinacolon Companies, respectively. ThT, ANS and other chemicals were obtained from Sigma‐Aldrich. The tau34‐pET21 construct, encoding the 412 amino acid tau protein,^[68]^ was kindly gifted by Professor Riazi (University of Tehran, Iran).

#### Tau Protein Expression and Purification

The *E.coli* BL21 (DE3) glycerol stock of the complementary DNA (c‐DNA) encoding the tau protein with a His‐tag (1 N4R isoform; GenBank No: P10636) in the pET‐21a (+) vector was used.^[68]^ Cultivation of the bacterial cells took place in 1 liter of Luria‐Bertani (LB) broth supplemented with 100 μg/ml ampicillin at 37 °C under agitation at 190 rpm. Upon induction with 0.5 mM IPTG at 37 °C for 4 hours, the cells were collected, suspended in threefold volumes of lysis buffer (50 mM Tris buffer, pH 7.8, 250 mM NaCl, 5 mM DTT, and 2 mM PMSF), and sonicated. Then it was centrifuged at 20,000 g for 30 minutes. The resultant supernatant was subjected to purification using Nickel Sepharose resin based on previously described methodology.^[46]^ In brief, the resin was initially equilibrated with a wash buffer (50 mM Tris buffer, pH 7.8, 250 mM NaCl, and 30 mM imidazole). This was then followed by the elution of the tau protein with a buffer solution containing 50 mM Tris buffer, pH 7.8, 250 mM NaCl, and 100 mM imidazole. Fractions containing the highest purity of the protein were pooled, concentrated, and dialyzed against 50 mM Tris buffer pH 7.4 and 100 mM NaCl. The concentration of tau protein was calculated using UV absorption at 280 nm with an extinction coefficient of 7450 M^−1^ cm^−1^. Finally, the purified tau protein was stored at −70 °C.

#### Incubation Conditions of Native Tau Samples in either the Presence or Absence of Metal Ions

Tau protein solutions at a concentration of 20 μM were prepared in 50 mM Tris buffer at pH 7.4 and 100 mM NaCl. These solutions were then subjected to the addition of 5 μM heparin (with an average molecular weight of 16 KDa), 5 mM DTT and varying concentrations (1 mM, 0.1 mM, and 0.01 mM) of Zn^2+^, Cu^2+^, and Fe^3+^ ions.^28^ The tau protein mixtures, along with heparin and DTT, were exposed to Zn^2+^, Cu^2+^, and Fe^3+^ ions separately at different molar ratios resulting in a molar ratio of 1 : 2, 1 : 5, and 1 : 50 for tau: metal ions. Following this, the samples were sealed in 20 mL bottles with a final volume of 1 mL each. Subsequently, the vials were placed on a shaker operating at 500 rpm and maintained at 37 °C for a duration of 96 hours to induce fibrillation. During the incubation period, 1 mM DTT was supplemented daily to the tau samples (after every 24 hours). The samples underwent identical agitation conditions. Furthermore, another tau sample lacking Zn^2+^, Cu^2+^, and Fe^3+^ ions (referred to as the not‐heated sample) was stored at 4 °C for 96 hours without agitation.

#### SDS‐PAGE Analysis

The protein samples were evaluated using sodium dodecyl sulfate polyacrylamide gel electrophoresis (SDS‐PAGE) using 12 % gels in reducing (with DTT) and non‐reducing conditions. The protein samples, 20 μg tau were loaded into the gel. Coomassie brilliant blue (CBB) dye was used to visualize the protein bands.

#### ThT and ANS Fluorescence Assay

The ThT fluorescence assay was performed using a Cary‐Eclipse spectrofluorometer at an excitation wavelength of 440 nm with a slit width of 10 nm and emission wavelengths of 400–650 nm with a slit width of 10 nm. Tau protein samples (0.2 mg/ml) in the presence of the metals were incubated with 25 μM ThT at room temperature for 10 min in the dark. To the tau protein samples, the ANS solution was added in the presence of metals at a final concentration of 100 μM. Tau protein samples had a concentration of 0.2 mg/ml. Incubation of tau protein with ANS was done at room temperature in the dark for 30 min. The fluorescence intensity of ANS‐bound tau protein was recorded by using Cary‐Eclipse spectrofluorometer with excitation set at 365 nm, while recording the emission from 400 to 600 nm.

#### Atomic Force Microscopy Analysis

The morphology of each sample was observed by quantitative atomic force microscopy (AFM) at the Institute of Biochemistry and Biophysics, University of Tehran, Iran. Tau protein samples (5 μL) were deposited onto freshly cleaved mica and left at room temperature for 30 minutes fixation. Subsequently, the surface of the mica was washed twice with 50 μL of deionized water and left to dry.

#### Far‐UV CD Spectroscopy Analysis

Far‐UV CD spectra were collected using an AVIV spectropolarimeter (Aviv Associates, Lakewood, NJ, USA), in the wavelength range of 190–260 nm. The experiments were carried out with a tau protein concentration of 0.2 mg/ml. The CD spectra of the blank solutions lacking tau protein were measured and subtracted from the CD spectra of tau‐treated samples with metals. All measured data were expressed as ellipticity versus wavelength. In addition, the secondary structure content of tau protein samples was calculated using the BeStSel online software.^[41]^

#### Cell Viability Assessment

Human neuroblastoma SH‐SY5Y cells were grown in high‐glucose DMEM containing 10 % FBS and kept at 37 °C in a humidified atmosphere (95 %) with 5 % CO_2_. The cells were seeded into a 96‐well culture plate at a density of 10,000 cells/well. Aliquots of the tau protein samples, including not‐heated, not‐treated and tau‐treated samples with Zn^2+^, Cu^2+^, and Fe^3+^ ions, were added to the SH‐SY5Y cells for 24 hours at 37 °C at a final tau concentration of 5 μM. Moreover, to study the neurotoxicity of the metals, each related blank sample was added to the cells at the same volumes as the tau‐treated samples. Cell viability was determined using the MTT assay. After treatment, cell medium was removed and replaced with the MTT solution (0.15 mg/ml in fresh medium) and incubated for 3 hours. The culture medium was then gently removed, and 100 μL of 10 % DMSO was added to the cells. Finally, the absorbance of the samples was measured at 570 nm by an ELISA reader spectrophotometer. Cell viability was then calculated in comparison with the negative treated control cells.

#### Quantification of Free Thiol Groups

Tau solutions with a final concentration of 0.86 mg/ml were incubated, then DTNB solution was added to achieve a tau: DTNB molar ratio of 1 : 7. Samples were incubated for an additional 90 minutes in the dark. Free thiol groups were quantified by measuring absorbance at 412 nm using a Thermo Nanodrop device, applying a molar extinction coefficient of 14150 M^−1^ cm^−1^ for thionitrophenylate anion at 412 nm.^[69]^

#### Data Analysis

Data were presented as mean ± SD. Statistical analysis was performed using GraphPad Prism 8.4.3, employing One‐way ANOVA with Dunnett's multiple comparisons test and t‐test to compare tau‐treated samples with the not‐heated and not‐treated samples. In all experiments, the blank samples lacking tau protein were incubated in either the presence or absence of metal ions, under the mentioned conditions.

## Nomenclature

AD: Alzheimer's disease
Aβ: Amyloid beta
AFM: Atomic force microscopy
ANS: 1‐Anilino‐8‐naphthalene sulfonate
APP: Amyloid precursor protein
CD: Circular dichroism spectroscopy
DTNB: 5,5′‐Dithio bis(2‐nitrobenzoic)
DTT: Dithiothreitol
IL‐1β: Interleukin 1 beta
IPTG: Isopropyl ß‐D‐1 thiogalactopyranoside
MPHD: Metal‐ Protein‐Heparin‐ Dithiothreitol
MTT: 3‐(4,5‐dimethylthiazol‐2‐yl)‐2,5‐diphenyltetrazolium bromide
NFT: Neurofibrillary tangle
PHD‐4 °C: Protein‐Heparin‐ Dithiothreitol‐4 degrees Celsius
PHD‐37 °C: Protein‐Heparin‐ Dithiothreitol‐37 degrees Celsius
ROS: Reactive oxygen species
SDS‐PAGE: Sodium Dodecyl Sulphate‐Polyacrylamide Gel Electrophoresis
TAU: Tubulin‐associated unit
ThT: Thioflavin‐T
TNF‐α: Tumor necrosis factor α

## Author Contributions

**Mahzad Irandoust**: Formal analysis, Investigation, Methodology, Data curation, Writing original draft. **Afrooz Anbaraki**: Formal analysis. **Zahra Dindar**: Formal analysis**. Atiyeh Ghasemi**: Data collection and methodology for MTT assay. **Ali Akbar Saboury**: Resources, Methodology and supervision for MTT assay. **Arefeh Seyedarabi**: Conceptualization, Funding acquisition, Methodology, Resources, Supervision, Review & editing.

## Conflict of Interests

The authors declare that there are no conflicts of interest with the contents of this article.

## Supporting information

As a service to our authors and readers, this journal provides supporting information supplied by the authors. Such materials are peer reviewed and may be re‐organized for online delivery, but are not copy‐edited or typeset. Technical support issues arising from supporting information (other than missing files) should be addressed to the authors.

Supporting Information

## Data Availability

The data that support the findings of this study are available in the supplementary material of this article.
