# Supplementary material for: Concentration‐Based Analysis of Metal‐Induced Tau Fibrillar versus non‐fibrillar Aggregation: Implications for Neurotoxicity in Alzheimer's Disease
Source: ChemistryOpen. 2025 Mar 24;14(8):e202400493. doi: 10.1002/open.202400493 (PMC12368877; doi:10.1002/open.202400493)
Supplement: Supplementary file 1 — Supporting Information [file OPEN-14-e202400493-s001.pdf]

ChemistryOpen

Supporting Information

Concentration-Based Analysis of Metal-Induced Tau Fibrillar versus non-fibrillar Aggregation: Implications for Neurotoxicity in Alzheimer's Disease

Mahzad Irandoust, Afrooz Anbaraki, Zahra Dindar, Atiyeh Ghasemi, Ali Akbar Saboury, Saeed Rayati, and Arefeh Seyedarabi*

Tables S1, S2, and S3. The percentage of secondary structure content of tau samples treated with different concentrations of Zn^{2+}, Cu^{2+}, and Fe^{3+}, respectively

Structure	PHD-4°C	MHPD 1mM ZN-4°C	PHD-37°C	MHPD 1mg/ml	MHPD 0.1mg/ml	MHPD 0.01mg/ml
Alpha-helix	10.8	8	1.9	7.6	9.5	0.7
Antiparallel	15.3	21.7	18	23	17.7	9.7
parallel	0	2.3	24.1	0.4	8	23
Beta-turn	13.9	15.2	14	16	14.3	19.9
Random-coil	60.1	52.8	42	53	50.6	46.8

Table 1S - Percentage of Secondary Structures for Protein Treatments with Zn^{2+}

Structure	PHD-4°C	MHPD 1mM Cu-4°C	PHD-37°C	MHPD 1mg/ml	MHPD 0.1mg/ml	MHPD 0.01mg/ml
Alpha-helix	10.8	6	1.9	4.2	12.3	9.7
Antiparallel	15.3	20	18	25.2	17.3	15.4
parallel	0	1.1	24.1	0.6	6.2	16.9
Beta-turn	13.9	14.1	14	17	11.9	9.6
Random-coil	60.1	58.7	42	53.1	52.2	41.4

Table 2S - Percentage of Secondary Structures for Protein Treatments with Cu^{2+}

Structure	PHD-4°C	MHPD 1mM Fe-4°C	PHD-37°C	MHPD 1mg/ml	MHPD 0.1mg/ml	MHPD 0.01mg/ml
**Alpha-helix**	10.8	6.4	1.9	5.9	6.2	10.6
**Antiparallel**	15.3	23.6	18	24	20.7	15.1
**parallel**	0	2.3	24.1	1.9	8	22.9
**Beta-turn**	13.9	14.4	14	14.8	11.6	6.9
**Random-coil**	60.1	53.2	42	53.5	53.4	44.5

Table 3S- Percentage of Secondary Structures for Protein Treatments with Fe^{3+}

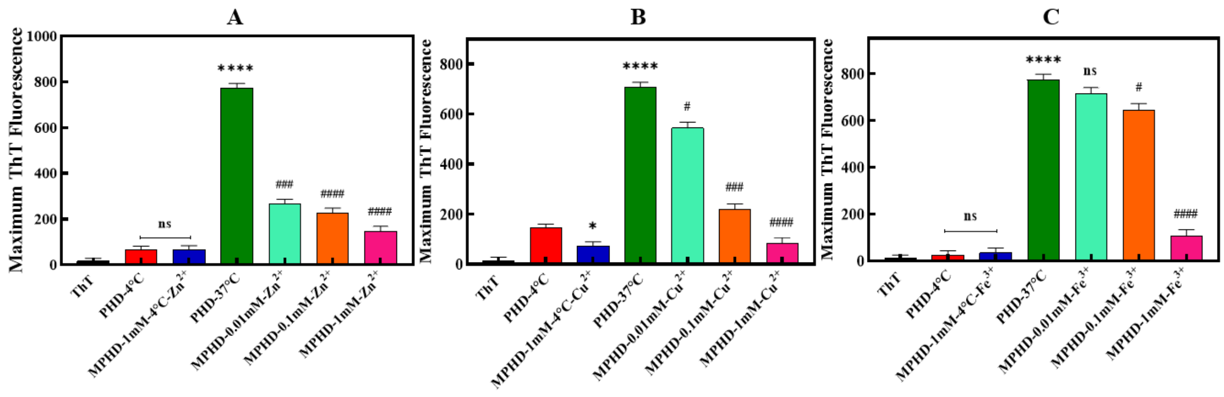

Figure S1. Statistical exploration of ThT fluorescence intensity in tau samples treated with varying metal concentrations. (A-C) Maximum ThT fluorescence intensity of different incubated samples of tau protein at 485 nm. Statistical analysis was performed by t-test and one-way ANOVA followed by Dunnett's multiple comparisons test. ***$P < 0.001$ and **$P < 0.01$, significantly different from the negative control (PHD-4°C)

sample. #P < 0.05, ##P < 0.01, ###P < 0.001, significantly different compared to the Positive control sample. (PHD: Protein + Heparin + DTT). (MPHD: Metal + Protein + Heparin + DTT). The samples, abbreviated as MPHD, were incubated at 37°C for 96 hours at three different concentrations of various metal ions, along with the positive control sample.

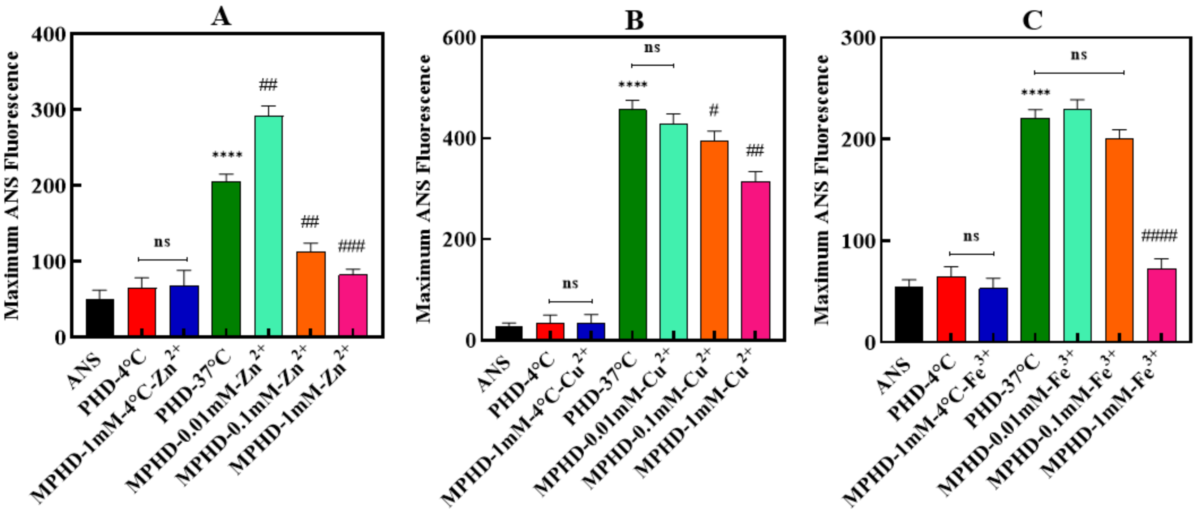

Figure S2. Statistical exploration of ANS fluorescence intensity in tau samples treated with varying metal concentrations. (A-C) Maximum ANS fluorescence intensity of different incubated samples of tau protein at 490 nm. Statistical analysis was performed by t-test and one-way ANOVA followed by Dunnett's multiple comparisons test. ***P < 0.001 and **P < 0.01, significantly different from the negative control (PHD-4°C) sample. #P < 0.05, ##P < 0.01, ###P < 0.001, significantly different compared to the Positive control sample. (PHD: Protein + Heparin + DTT). (MPHD: Metal + Protein + Heparin + DTT). The samples, abbreviated as MPHD, were incubated at 37°C for 96 hours at three different concentrations of various metal ions, along with the positive control sample.

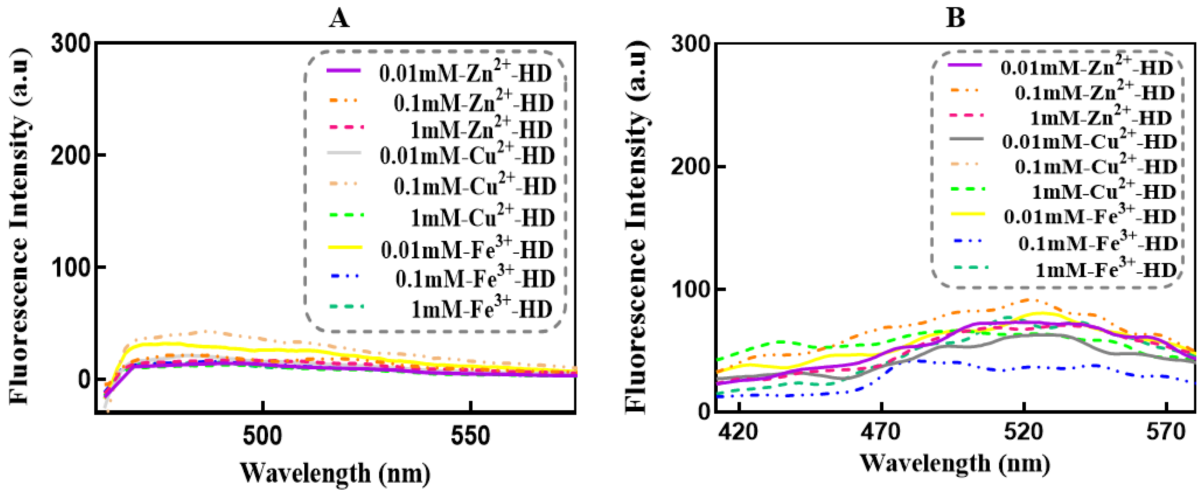

Figure S3. The fluorescence emission spectra of ThT and ANS for metal-containing samples devoid of tau protein. (A) ThT fluorescence emission spectra of the samples lacking tau protein that were treated with different concentrations of various metals, respectively. (B) ANS fluorescence emission spectra of the samples lacking tau protein that were treated with different concentrations of various metals, respectively.(HD: Heparin+DTT). All samples were incubated for 96 hours at 37°C.

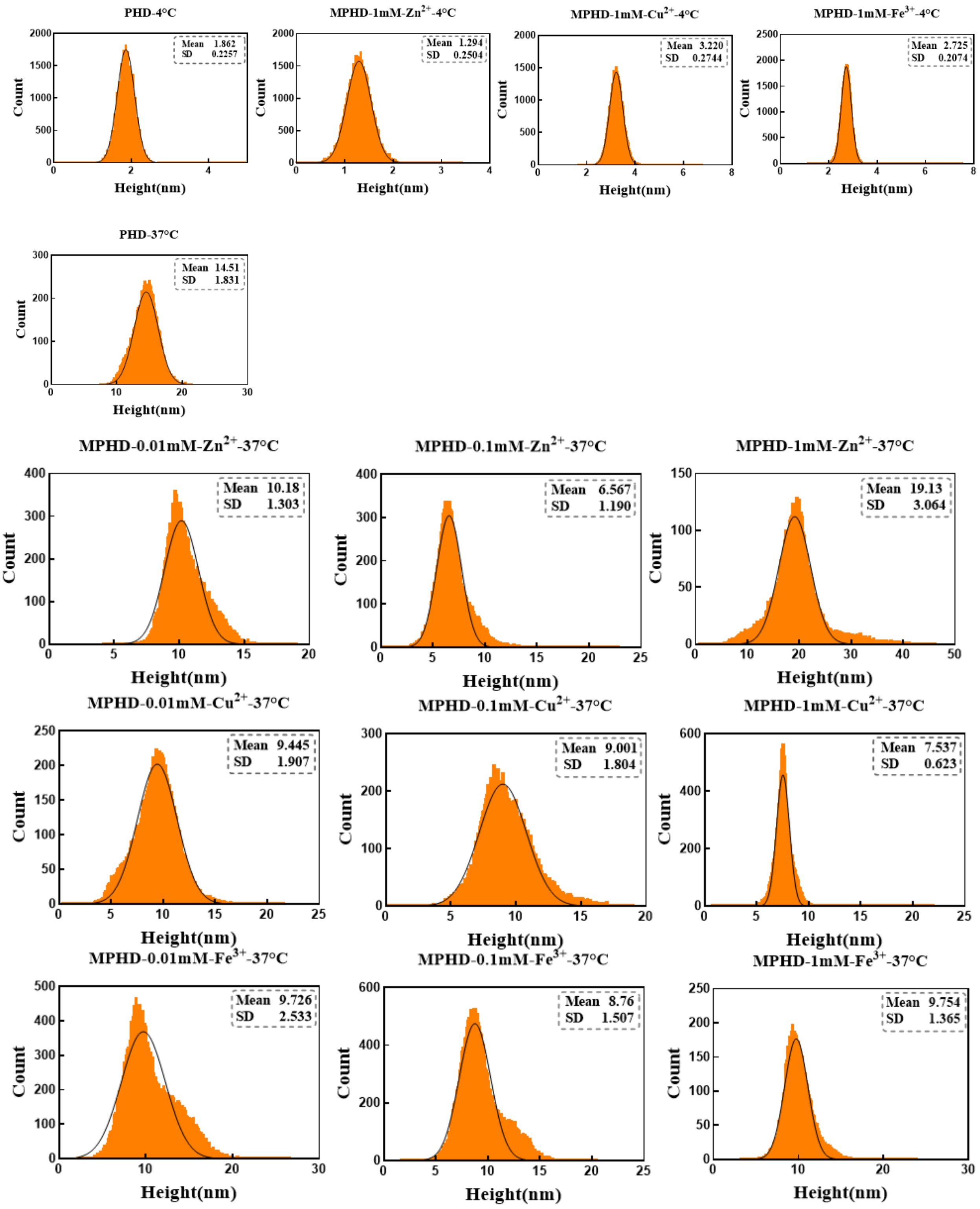

Figure S4. Histogram illustrating the height distribution function derived from atomic force microscopy (AFM) images of native tau samples subjected to treatment with various metals.

The mean height of the samples is represented within these graphical depictions. (PHD: Protein + Heparin + DTT). (MPHD: Metal + Protein + Heparin + DTT).

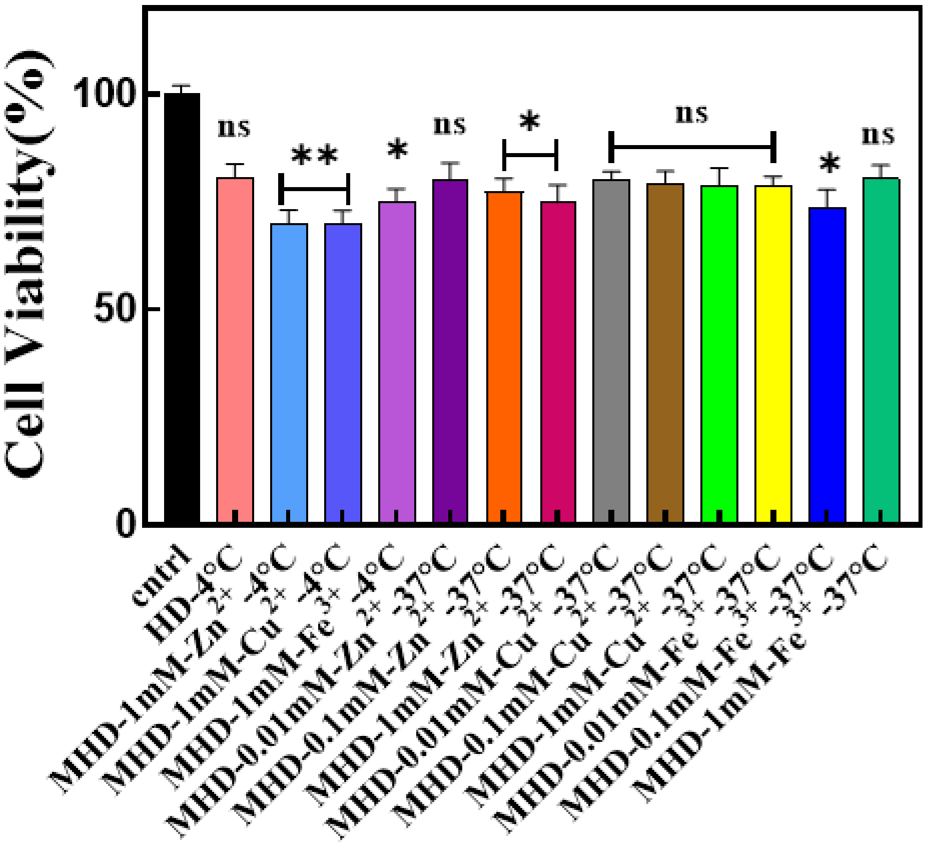

Figure S5. Cell viability assay of the effects of different concentrations of different metals alone in the absence of tau protein on SH-SY5Y cells. Cell viability values are expressed as mean ± SD. Statistical analysis was performed using t-test and one-way analysis of variance followed by Dunnett's multiple comparison test (* $p < 0.05$, ** $p < 0.01$, *** $p < 0.001$). (HD: Heparin+DTT).(MHD: Metal+ Heparin+ DTT).
